# Heterogeneity in the Infection Biology of *Campylobacter jejuni* Isolates in Three Infection Models Reveals an Invasive and Virulent Phenotype in a ST21 Isolate from Poultry

**DOI:** 10.1371/journal.pone.0141182

**Published:** 2015-10-23

**Authors:** Suzanne Humphrey, Lizeth Lacharme-Lora, Gemma Chaloner, Kirsty Gibbs, Tom Humphrey, Nicola Williams, Paul Wigley

**Affiliations:** 1 Department of Infection Biology, Institute of Infection and Global Health, University of Liverpool, Leahurst Campus, Neston, United Kingdom; 2 Department of Epidemiology and Population Health, Institute of Infection and Global Health, University of Liverpool, Leahurst Campus, Neston, United Kingdom; 3 School of Veterinary Science, University of Liverpool, Leahurst Campus, Neston, United Kingdom; Leibniz Institute for Natural Products Research and Infection Biology- Hans Knoell Institute, GERMANY

## Abstract

Although *Campylobacter* is the leading cause of bacterial foodborne gastroenteritis in the world and the importance of poultry as a source of infection is well understood we know relatively little about its infection biology in the broiler chicken. Much of what we know about the biology of *Campylobacter jejuni* is based on infection of inbred or SPF laboratory lines of chickens with a small number of isolates used in most laboratory studies. Recently we have shown that both the host response and microbial ecology of *C*. *jejuni* in the broiler chicken varies with both the host-type and significantly between *C*. *jejuni* isolates. Here we describe heterogeneity in infection within a panel of *C*. *jejuni* isolates in two broiler chicken breeds, human intestinal epithelial cells and the *Galleri*a insect model of virulence. All *C*. *jejuni* isolates colonised the chicken caeca, though colonisation of other parts of the gastrointestinal tract varied between isolates. Extra-intestinal spread to the liver varied between isolates and bird breed but a poultry isolate 13126 (sequence type 21) showed the greatest levels of extra-intestinal spread to the liver in both broiler breeds with over 70% of birds of the fast growing breed and 50% of the slower growing breed having *C*. *jejuni* in their livers. Crucially 13126 is significantly more invasive than other isolates in human intestinal epithelial cells and gave the highest mortality in the *Galleria* infection model. Taken together our findings suggest that not only is there considerable heterogeneity in the infection biology of *C*. *jejuni* in avian, mammalian and alternative models, but that some isolates have an invasive and virulent phenotype. Isolates with an invasive phenotype would pose a significant risk and increased difficulty in control in chicken production and coupled with the virulent phenotype seen in 13126 could be an increased risk to public health.

## Introduction

*Campylobacter* spp. are considered to be the leading cause of bacterial foodborne gastroenteritis in the world. In the EU alone there are considered to be in excess of 200,000 confirmed human cases per annum, although these are likely to represent a small proportion of actual cases [1]. *Campylobacter jejuni* is associated with the majority of human infections, accounting for approximately 80% of cases in the EU [1].

Poultry products are considered to be the most significant source of human campylobacteriosis, with up to 80% of fresh broiler meat contaminated with *Campylobacter* spp. at the point of retail sale [1]. Much of this contamination is believed to occur during the evisceration stage of the slaughter process, when gut contents containing up to 10^9^ *Campylobacter* cells per gram in colonised birds may contaminate carcasses on the production line. Surface treatment of carcasses, using chlorine or lactic acid sprays, for example, represents a central intervention strategy for controlling *Campylobacter* in the food chain [2, 3]. Importantly, however, *C*. *jejuni* is also capable of extra-intestinal spread from the chicken intestine into edible tissues such as the liver and deep muscle [4–10], which has significant implications for public health, as bacteria within these sites cannot be eliminated using surface treatments and may better survive under-cooking.

Despite the importance of the broiler chicken as the main source of human *C*. *jejuni* infection of the broiler chicken remains relatively poorly understood. *Campylobacter* had been considered a normal harmless component of the commensal intestinal microbiota in the chicken. However, increasing evidence suggests that *Campylobacter* is capable of inducing intestinal damage at a cellular level by compromising intracellular tight-junctions and modulating the barrier function of the intestinal epithelia [11, 12], and indirectly through stimulation of poorly-regulated host inflammatory responses [13]. In addition, a link has been suggested between the presence of *Campylobacter* in poultry flocks and increased incidence of the leg pathologies pododermatitis and hock burn [10, 14]. Our current understanding of the infection biology of *Campylobacter* in the chicken is derived from studies using a limited number of *Campylobacter* strains in specified pathogen free flocks consisting of inbred lines or slow-growing traditional breeds that are not necessarily representative of the modern fast-growing commercial broiler chicken. Furthermore our recent work has shown that the breed of chicken has a significant impact on the host response to infection [15] and that there is variation both in extra-intestinal spread and colonisation of the gastrointestinal tract between *C*. *jejuni* isolates [16]

It is not clear to what extent heterogeneity between *C*. *jejuni* isolates impacts on human infection. Indeed the absence of a good animal model for *Campylobacter* gastroenteritis has left considerable gaps in our understanding. A number of approaches including the use of cell-based models, tissue explants and invertebrate models have been utilised to assess the virulence of *C*. *jejuni* to varying degrees of success. Human epithelial cell lines are a good indicator of the ability of bacterial isolates to invade the gut epithelium and have been used extensively in the study of a number of enteric pathogens [17]. Infection of the larvae of the Wax Moth (*Galleria mellonella*), sometimes called the wax worm, has been used as a measure of *Campylobacter* virulence [18–20]. Measurement of mortality following inoculation of bacteria into the haemolymph of the larvae can assess both the relative virulence of isolates and the role of putative virulence factors in pathogenesis.

In this work we aimed to compare the infection biology of a small panel of *C*. *jejuni* isolates in three infection models; *in vivo* in the broiler chicken, *in vitro* in Caco2 human epithelial cells and finally in *Galleria* larvae. Two breeds of chicken were used to ascertain differences in infection between isolates in order to establish the effect of *Campylobacter* strain and broiler breed in influencing the ecology of intestinal colonisation and extra-intestinal spread to edible tissues. Caco-2 cells allow us to assess variation in invasive ability, and *Galleria* larvae as a measure of general virulence. Our findings show considerable heterogeneity in infection biology between isolates and that certain *C*. *jejuni* isolates show marked differences in infection ecology, invasion and virulence that has implications for public health.

## Materials and Methods

### Bacterial strains and culture conditions

All strains used in this work are described in Table 1. Bacteria were grown from stocks maintained at -80°C on Columbia Blood Agar (Lab M, Heywood, Lancashire, UK) supplemented with 5% defibrinated horse blood (Oxoid, Basingstoke, Hampshire, UK) for 48h in microaerobic conditions (80% N_2_, 12% CO_2_, 5% O_2_ and 3% H_2_) at 41.5°C. Liquid cultures were grown for 24h in 10 ml of Mueller-Hinton broth (MHB) (Lab M, Heywood, Lancashire, UK) in microaerobic conditions at 41.5°C.

**Table 1:** *C*. *jejuni* strain panel.

Strain	Source	Sequence Type/Clonal Complex
M1*	Human	ST137/CC45
13126^†^	Poultry	ST21/CC21
12662^†^	Poultry	ST257
DBM1^‡^	Poultry; deep breast muscle tissue	ST3444
NCTC 11168H^§^	Laboratory strain	ST43/CC21

Strains kindly provided by

*Dr Lisa Williams, University of Bristol

^†^Food Standards Agency, UK

^‡^Dr Camilla Brena, University of Liverpool

^§^Dr Nick Dorrell, London School of Hygiene and Tropical Medicine.

### Experimental animals

All work was conducted in accordance with UK legislation governing experimental animals under Home Office project licence 40/3652 and was approved by the University of Liverpool ethical review process prior to the award of the licence. All animals were checked a minimum of twice daily to ensure their health and welfare. Two commercial broiler breeds were used. Data from the breeding companies state that the A bird reaches live slaughter weight (2.2 Kg) at 36 days of age, while the B bird takes 56 days.

Age-matched, 1-day-old mixed sex broiler chicks were obtained from a commercial hatchery. Chicks were maintained in floor pens at Home Office (UK government) recommended stocking levels and were given *ad libitum* access to water and a pelleted vegetable protein-based diet (SDS, Witham, Essex, UK). Chicks were housed in separate groups of 10 or 11 animals, with each breed housed in a separate room. Rooms were maintained at a temperature of 30°C, which was reduced to 20°C when the birds were 3 weeks of age. Prior to experimental infection, all birds were confirmed as *Campylobacter*-free by taking cloacal swabs, which were streaked onto selective blood-free agar (mCCDA) supplemented with *Campylobacter* Enrichment Supplement (SV59; Mast Group, Bootle, Merseyside, UK) and grown for 48h in microaerobic conditions at 41.5°C. The use of vaccination in broiler-breeder flocks and strict hygiene and biosecurity guidelines set down by legislation in National Control Plans have virtually eliminated *Salmonella* from UK hatcheries and so the risk of co-infection is minimal.

### Infection of chickens

All work was conducted in accordance with UK legislation governing experimental animals under project licence 40/3652 and was approved by the University of Liverpool ethical review process prior to the award of the licence. All animals were checked a minimum of twice daily to ensure their health and welfare.

At 21 days old, A (n = 11) and B (n = 10 or 11) broilers were orally infected with 2 x 10^5^ cells of *C*. *jejuni* strains M1, 13126, 12662 or DBM1 suspended in 0.2 ml of MHB. At 4 dpi, the cloaca of all birds was swabbed to monitor *C*. *jejuni* colonisation and shedding. At 11 dpi, all birds were killed by overdose of anaesthetic. At post-mortem examination, samples of breast muscle and liver tissue and gut contents were taken aseptically and processed for *Campylobacter* enumeration. NCTC1168H was not included in these studies for logistical reasons and has previously been well-characterised in the chicken by us and others [21–23]

### Assessment of *C*. *jejuni* intestinal load

As a crude measure of colonisation, determination of faecal shedding of *C*. *jejuni* at 4DPI was carried out using semi-quantitative approach to enumeration from cloacal swabs [24, 25]. Briefly, cloacal swabs were taken and eluted in 2 ml modified Exeter broth consisting of 1100 ml nutrient broth (Lab M, Heywood, Lancashire, UK), 11ml lysed defibrinated horse blood (Oxoid, Basingstoke, Hampshire, UK), 10 ml *Campylobacter* Enrichment Supplement SV59 (Mast Diagnostics), and 10 ml *Campylobacter* Growth Supplement SV61 (Mast Diagnostics). Swabs were then plated onto mCCDA agar supplemented with SV59. Enriched swabs were incubated at 41.5°C for 48h before re-plating onto mCCDA agar supplemented with SV59. Plates were incubated for 48h at 41.5°C under microaerobic conditions before being scored for the level of bacterial growth. Bacterial growth was recorded as both number and percentage of birds falling into each of three levels of colonisation (Heavy (H)>50 colonies by direct plating, Direct (D) detections by direct plating onto mCCDA and Total (T) total where *Campylobacter* detected by direct plating or following enrichment). The total (T) includes both positive from H and D colonisation along with those obtained from enrichment culture.

In order to determine the levels of gut colonisation by each *C*. *jejuni* strain in each of the breeds, ileal and caecal contents were collected from individual birds at necropsy and diluted in 9 volumes of maximal recovery diluent (MRD). Serial 10-fold dilutions were made of each sample in MRD and based upon the method of Miles and Misra, duplicate 50 μl spots were plated onto mCCDA agar supplemented with SV59. Plates were incubated as above. *C*. *jejuni* colonies were counted to give CFU/g of gut contents.

### Assessment of *C*. *jejuni* extra-intestinal spread

In order to determine the presence of *C*. *jejuni* in the edible tissues of each of the broiler breeds, liver and breast muscle tissue were collected aseptically from individual birds at necropsy. Liver tissue samples were homogenised in 3-5ml MRD and streaked onto mCCDA agar. Plates were incubated under microaerobic conditions at 41.5°C for 48h and non-quantitatively scored for the presence or absence of *Campylobacter* colonies.

To assess deep contamination of breast meat, the entire muscle was removed from the bird and was surface-sterilised by immersion in 100% (v/v) ethanol followed by flaming. Five sections of surface-sterilised breast muscle were cut using a flamed 7mm cork borer, homogenised in 2-3ml of MRD and streaked onto mCCDA agar. Plates were incubated under microaerobic conditions at 41.5°C for 48h and non-quantitatively scored for the presence or absence of *Campylobacter* colonies.

For enrichment of both liver and breast muscle, 100μl of each homogenised sample was used to inoculate 2ml Exeter enrichment broth and following incubation under microaerobic conditions at 41.5°C for 48h, were streaked onto mCCDA as before. After 48h, enrichment plates were scored for the presence or absence of *Campylobacter* colonies.

### Invasion in Caco-2 human colonic epithelial cells

Caco-2 cells were grown in 72 cm^2^ flasks (Cellstar, Greiner Bio-one, Frickenhausen, Germany) at 37°C in a humidified atmosphere consisting of 5% CO_2_/95% air. The growth medium comprised Dulbecco’s Modified Eagle’s Medium (DMEM) (Sigma) supplemented with 10% (v/v) foetal calf serum (Sigma), 1% (v/v) non-essential amino acids (Sigma), 1% (v/v) _L_-glutamine (Sigma) and 1% (v/v) Pen/Strep (Sigma).

For infection, Caco-2 cells were seeded at 1 x 10^5^ cells per well in a 24 well plate (Cellstar) and incubated at 37°C, 5% (v/v) CO_2_/95% air for 3 days until confluent. Twenty-four hours before infection, cells were washed three times with PBS and overlaid with 1ml antibiotic-free culture medium. Liquid cultures of each *C*. *jejuni* strain were adjusted to OD_600_ 0.10–0.13 in Mueller-Hinton broth to give approximately 10^8^ bacteria per ml. 100μl of adjusted *C*. *jejuni* culture was added to each well to give and approximate multiplicity of infection (MOI) 10, and infection was allowed to proceed for 4h at 37°C in 5% CO_2_.

Following infection, cells were washed three times with sterile PBS and overlaid with 1ml DMEM supplemented with 100μg gentamicin ml^-1^ (Sigma) for 1h at 37°C in 5% CO_2_. Cells were washed twice with sterile PBS and lysed by addition of 1ml 1% (v/v) Triton X100 in PBS for 5–10 minutes. Intracellular *C*. *jejuni* were quantified by serial 10-fold dilutions in MRD and,triplicate 20μ spots were plated onto CBA agar. Plates were incubated as above and the resulting colonies enumerated. The rate of invasion for each strain was expressed as a percentage of the bacterial inoculum at the start of the experiment ± standard deviation calculated from triplicate assays.

### *Galleria* infection

*G*. *melonella* infection was performed based on the method described by Gundogdu et al. [19]. Briefly, ten final instar *G*. *mellonella* larvae (2–3 cm long weighing 180–250 mg each; Live Foods Direct, United Kingdom) were inoculated with 10^6^CFU/10μl of an overnight culture of each *C*. *jejuni* isolate by microinjection into the haemocoel using a Hamilton Syringe (Hamilton, Switzerland). The larvae were incubated at 37°C, and mortality and appearance at 24 and 48 hours were recorded; data shown are mortality after 48 hours. *G*. *mellonella* larvae receiving injections of PBS and no-injection controls were included. Experiments were repeated 6 times.

### Statistical analyses

Statistical analyses were performed using SPSS v21 (IBM). For comparison of the effect of broiler breed on colonisation by a particular *C*. *jejuni* strain, the Mann Whitney U test was applied, as the data were not normally distributed. For comparison of the virulence of multiple *C*. *jejuni* strains, the Kruskal-Wallis non-parametric test was applied. For *in vitro* invasion of Caco-2 cells and *G*. *mellonella* infection, a one-way ANOVA with Tukey post-hoc test was used to determine statistically significant differences. In all tests, differences were considered significant where *p*<0.05.

## Results

### *C*. *jejuni* colonisation of the chicken intestinal tract

Four days after inoculation with *C*. *jejuni*, cloacal swabbing was performed on all birds in order to establish whether colonisation had occurred, as determined by shedding of the bacterium. Heavy shedding of strains 12662 and DBM1 was detected in almost all birds of both breeds (Table 2), indicating high-level early intestinal colonisation by these strains. *C*. *jejuni* M1 shedding was directly detectable in 9/11 infected breed A birds and 7/10 breed B birds, suggesting that this strain was less efficient than the field ones in colonising broilers. Following enrichment, M1 was detected in all birds of both breeds. Interestingly, while considerable *C*. *jejuni* 13126 shedding was detected by direct plating in all breed B birds at 4DPI, this strain was not detectable in any of the breed A birds at the same time point. This finding remained consistent in breed A birds following enrichment of swabs., This suggests that that establishment of 13126 colonisation is a more prolonged process in this breed. Furthermore, this is consistent with the colonisation dynamics we previously reported for 13126 and M1 in a dual infection experimental system [16].

**Table 2:** Colonisation dynamics of *C*. *jejuni* strains in two commercial broiler lines at 4DPI.

Strain	Broiler breed A			Broiler breed B		
	H	D	T	H	D	T
*C*. *jejuni* M1	6	3	11	3	4	10
	(54.5%)	(27.3%)	(100%)	(30%)	(40%)	(100%)
*C*. *jejuni* 13126	0	0	0	9	1	10
				(90%)	(10%)	(100%)
*C*. *jejuni* 12662	11 (100%)	0	11 (100%)	10 (90.9%)	1 (9.1%)	11 (100%)
*C*. *jejuni* DBM1	10	1	11	11	0	11
	(90.9%)	(9.1%)	(100%)	(100%)		(100%)

Cloacal colonisation of birds with *C*. *jejuni* strains M1, 13126 12662 and DBM1 at 4 DPI in birds infected at 21 days of age. Results are expressed as both number and percentage of birds falling into each of three levels of colonisation (Heavy (H)>50 colonies by direct plating, Direct (D) detections by direct plating onto mCCDA and Total (T) total where *Campylobacter* detected by direct plating or following enrichment). The total (T) includes both positive from H and D colonisation along with those obtained from enrichment culture.

**Fig 1:**
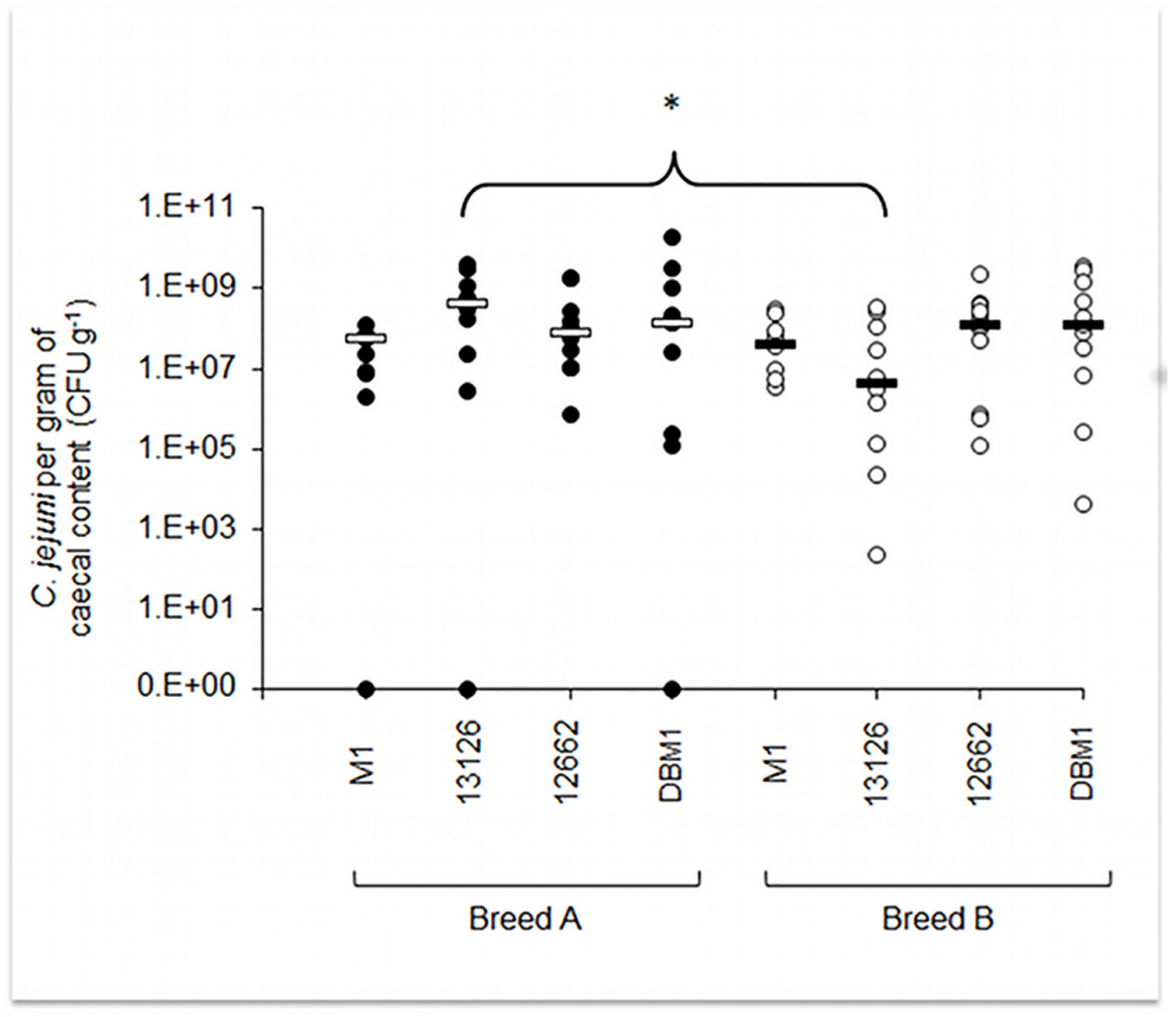
Caecal colonisation of two commercial broiler lines at 11DPI. Caecal colonisation of two broiler lines, A (closed circles) and B (open circles) by four *C*. *jejuni* strains. Bars represent the median value for each group. Asterisks show statistically significant differences in colonisation levels between the two broiler breeds as assessed by Mann Whitney U test (* p = 0.0024).

At 11DPI, all birds were killed and samples of caecal and ileal contents taken for determination of intestinal *C*. *jejuni* load. All *C*. *jejuni* strains tested were equally (*p*>0.05) able to colonise the caeca of both broiler breeds to a high level following experimental infection, with up to 1.83×10^10^ CFU per gram of content (Fig 1). However, *C*. *jejuni* could not be detected in one breed A bird in each of the M1, 13126 and DBM1 groups. Broiler breed did not have a significant (*p*>0.05) effect on caecal colonisation with *Campylobacter* strains M1, 12662 and DBM1. In the case of *C*. *jejuni* 13126, however, colonisation of the caeca of breed A birds was significantly (*p* = 0.024) higher than that in breed B birds (Fig 1).

**Fig 2:**
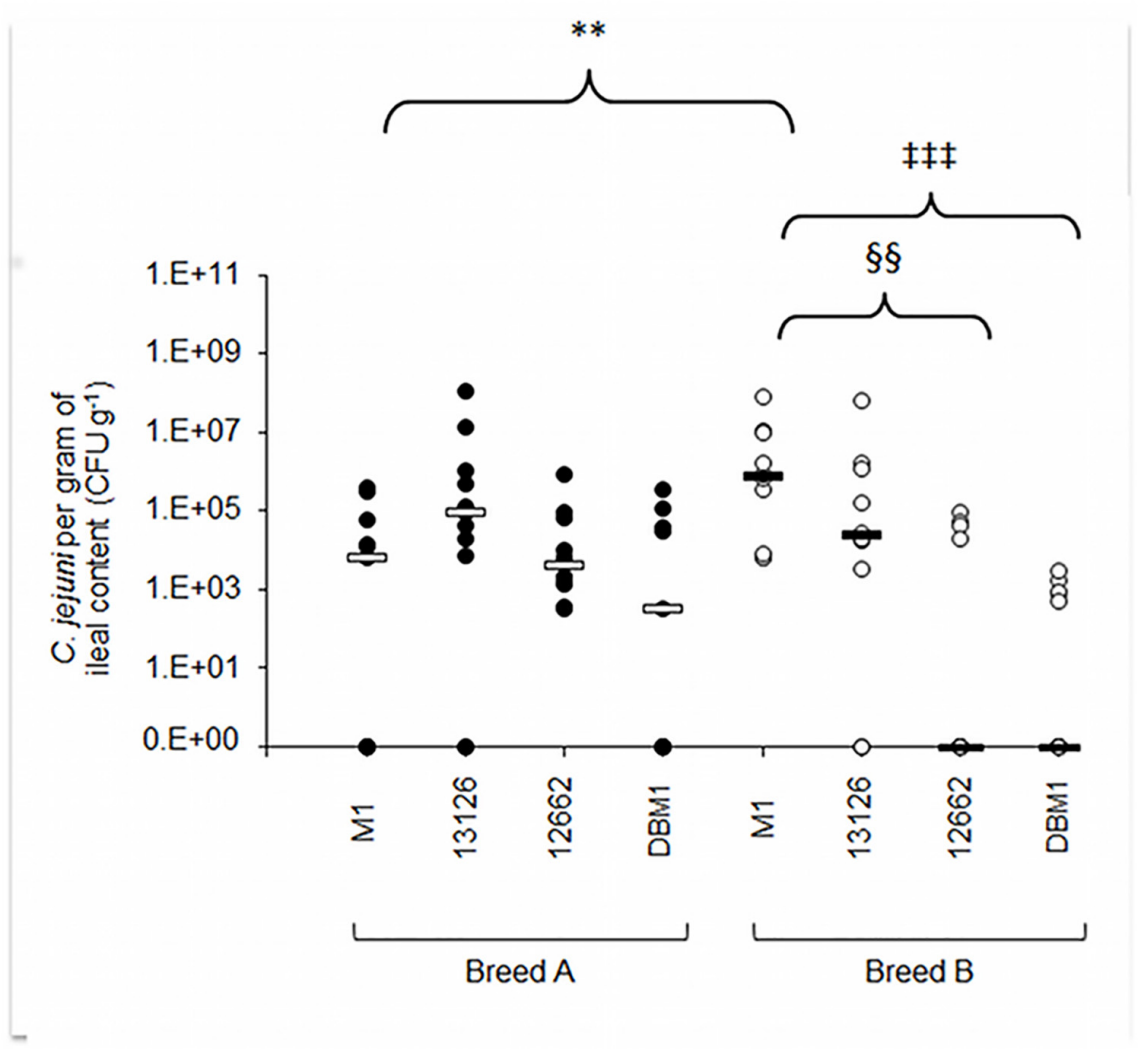
Ileal colonisation of two commercial broiler lines at 11DPI. Ileal colonisation of two broiler lines, A (closed circles) and B (open circles) by four *C*. *jejuni* strains. Bars represent the median value for each group. Asterisks show statistically significant differences in colonisation levels between the two broiler breeds as assessed by Mann Whitney U test (** p = 0.079; § p = 0.006; ‡ p = 0.001).

In the ileum, no significant differences (*p*>0.05) were found between the strains in terms of their ability to colonise breed A birds (Fig 2). M1 colonised the ileum of breed B birds to a significantly higher level than 12662 (*p* = 0.006) and DBM1 (*p* = 0.001), while 13126 colonised breed B birds to higher levels than DBM1, but this was not statistically significant (*p* = 0.052). In the case of *C*. *jejuni* M1, broiler breed had a significant (*p* = 0.0079) effect on the levels of ileum colonisation, with median bacterial load of this strain approximately two logs higher in breed B birds than in breed A (Fig 2). No effect of broiler breed on ileum colonisation was observed for the other *Campylobacter* strains.

### Extra-intestinal spread of *C*. *jejuni* to liver and muscle

Of the four *C*. *jejuni* strains tested, all demonstrated a capacity for spread to the liver, although the frequency of liver contamination varied both between the strains and by broiler breed (Table 3). In breed A birds, infection with 13126 or 12662 was associated with a high frequency of *Campylobacte*r detection in the liver following enrichment, with bacteria isolated from over 70% of livers in each group. These rates were significantly higher than for the other strains, M1 (*p* = 0.014 and *p* = 0.026, respectively) and DBM1 (*p* = 0.003 and *p* = 0.006, respectively). Previously we have reported a similar trend in liver invasion frequencies for 13126 and M1 during a dual infection experiment, with 13126 consistently exhibiting a higher frequency of extra-intestinal spread than M1[16]. The frequency of *C*. *jejuni* culture from the liver following experimental infection also varied between the strains in breed B birds but these differences were not statistically significant. Broiler breed did appear to have some effect on liver infection, with both M1 and DBM1 exhibiting higher levels of liver contamination in breed B birds than in breed A ones, while the rate of 13126 infection in the liver was reduced from 72.7% to 50% in breed B broilers. The most marked difference in the behaviour of a strain between the breeds was observed with 12662, where breed A birds had significantly (*p* = 0.016) higher levels of liver positivity than breed B ones. The underlying reasons for these observations are unclear at this time although could be related in differences in host response previously shown between these breeds.

**Table 3:** Extra-intestinal spread of *C*. *jejuni* to edible tissues at 11 DPI.

	Liver		Breast muscle	
Strain	Breed A	Breed B	Breed A	Breed B
*C*. *jejuni* M1	1/11 (9.1%)	4/10 (40%)	0/11 (0%)	0/10 (0%)
*C*. *jejuni* 13126	8/11 (72.7%)	5/10 (50%)	0/11 (0%)	0/10 (0%)
*C*. *jejuni* 12662	7/10 (70%)	1/11 (9.1%)	0/11 (0%)	0/11 (0%)
*C*. *jejuni* DBM1	0/11 (0%)	2/11 (18.2%)	0/11 (0%)	1/11 (9.1%)

Finally, contamination of deep muscle tissue was detected in one breed B bird in the group inoculated with DBM1 (Table 3). This observation suggests that although muscle contamination *in vivo* may be a rare event, it confirms there is potential for invasion of edible muscle tissue.

### *C*. *jejuni* exhibit heterogeneity in Caco-2 cell invasion

**Fig 3:**
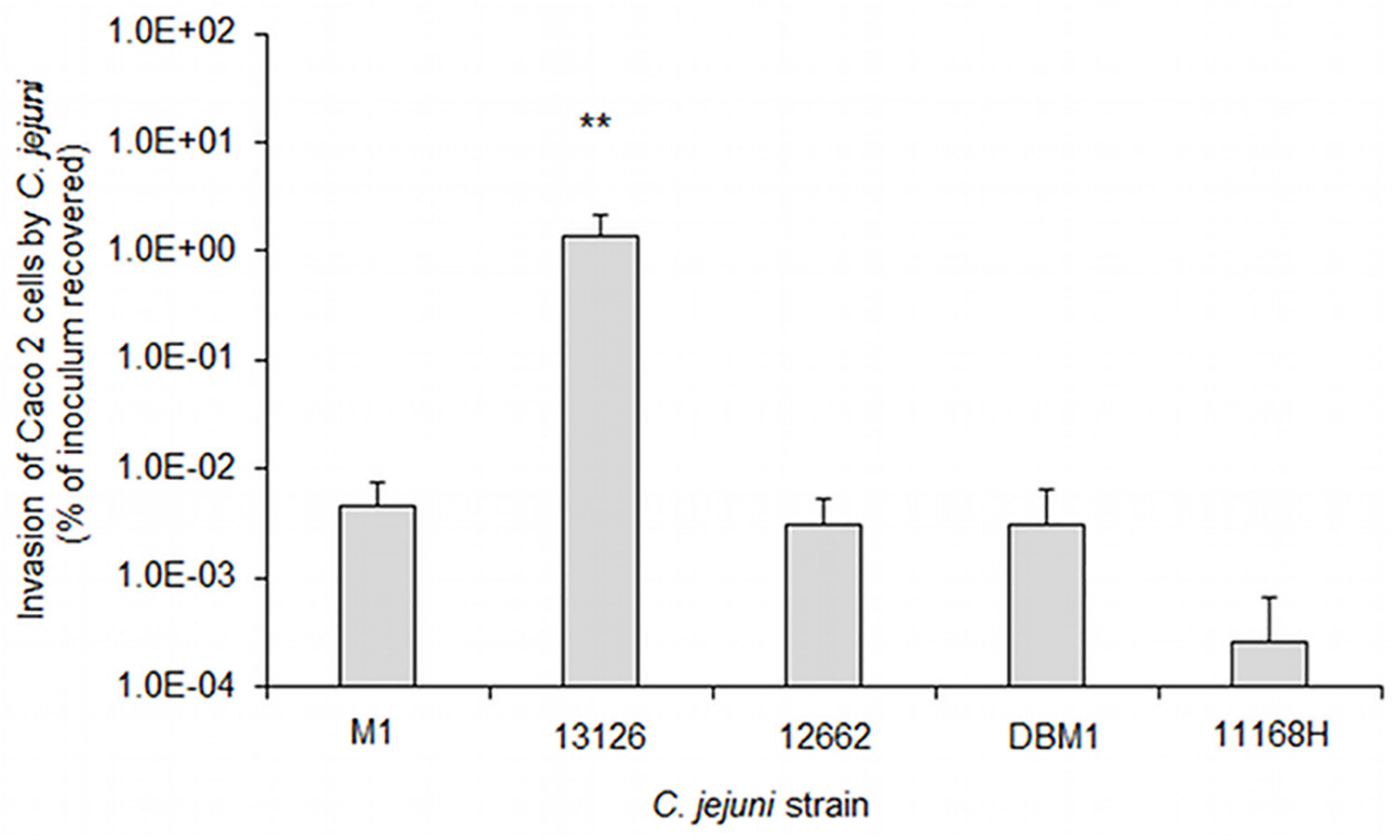
*C*. *jejuni* 13126 displays enhanced invasiveness in Caco-2 cells. Invasion of Caco 2 cells by five *C*. *jejuni* strains was examined. Asterisks show statistically significant differences in invasion levels between *C*. *jejuni* 13126 and the other four strains as assessed by one-way ANOVA (** p = 0.01).

All five *C*. *jejuni* strains tested exhibited some invasion of the Caco-2 cell line, although there was considerable variation in their invasive phenotypes (Fig 3). Notably, *C*. *jejuni* 13126 displayed significantly (*p* = 0.006) higher levels of invasion (1.38% of inoculum) than the other strains tested (all ≤0.005% of inoculum).

### *C*. *jejuni* leads to differential mortality rates in *Galleria* infection

**Fig 4:**
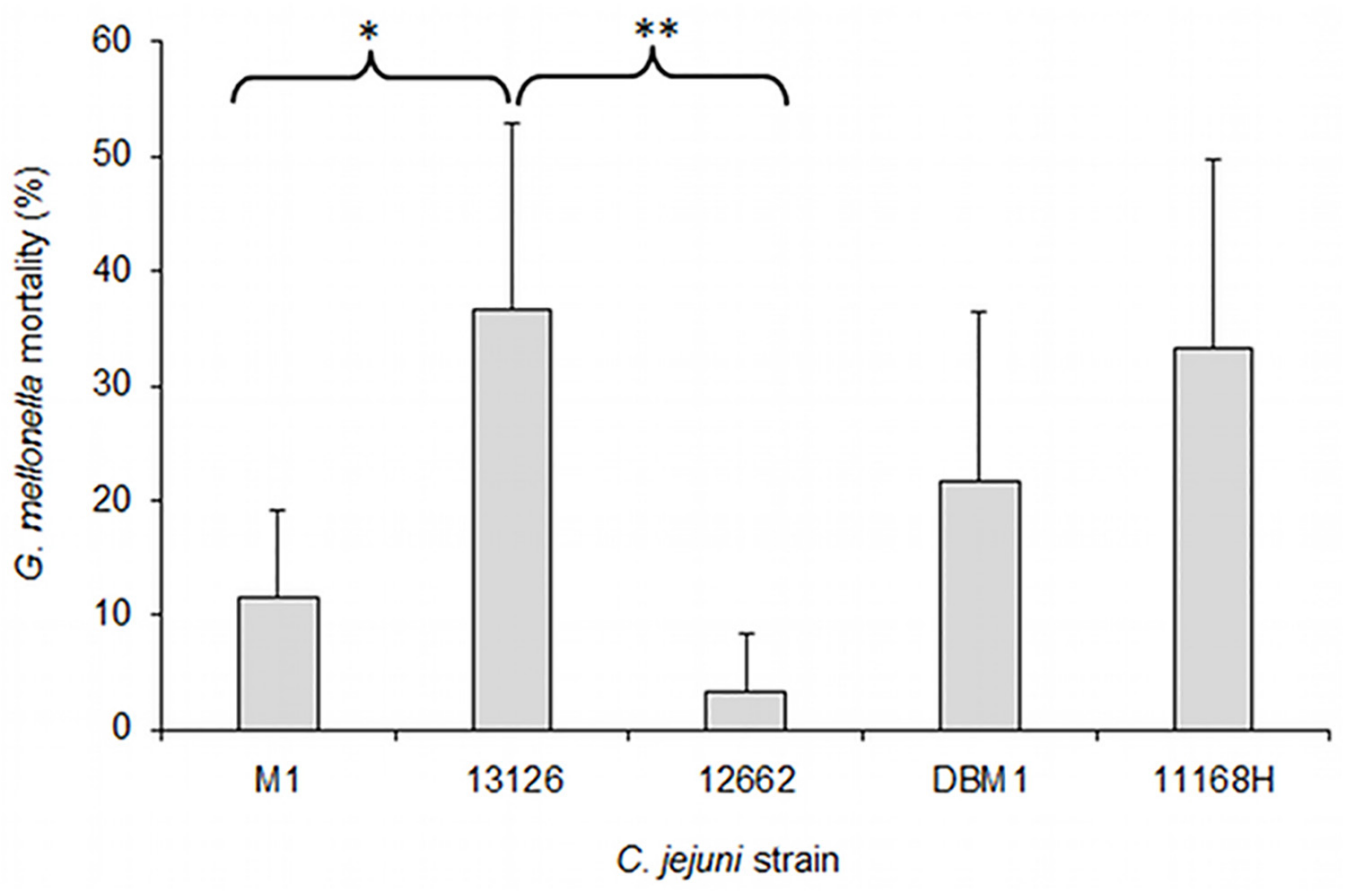
Mortality of *Galleria mellonella* larvae at 48h post infection with *C*. *jejuni*. Mortality as measured by larval viability 48 hours after challenge. Data presented based on six repeats of infection experiment using 10 larvae per isolate. Asterisks show statistically significant differences in mortality rate between *C*. *jejuni* 13126 and other strains as assessed by one-way ANOVA with Tukey post-hoc test (* p = 0.02; ** p = 0.001).

Mortality rates in *Galleria* larvae varied following infection by *C*. *jejuni* isolates (Fig 4). The greatest mortality rate was found for 13126 (36.6 ± 16.3), followed by the NCTC11168H (33.3 ± 16.3) and DBM1 (21.6 ± 14.7) isolates; there were no statistically significant differences between these three strains. Mortality rate for the 13126 isolate was significantly higher than that of the M1 and 12262 strains (11.6 ± 7.5 and 3.3 ± 5.2; *p* = 0.02 and *p* = 0.001, respectively). Less than 2% mortality was observed in the control groups. Interestingly, melanisation of the larvae infected with 13126 was observed within 30 minutes inoculation, whereas this took several hours for other isolates. Healthy *G*. *mellonella* larvae are a cream colour, but darken in response to the presence of a pathogen in their haemolymph; this is because when their immune system recognises a pathogen the activation of the phenoloxidase cascade leads to the melanisation of haemolymph around the pathogen[26]. This is indicative of a rapidly invasive phenotype in 13126

## Discussion

Here we show that there is considerable variation between *C*. *jejuni* isolates in differing infection models. Isolate 13126, a ST21 isolate that shows the greatest ability to spread from the chicken gastrointestinal tract is also highly invasive in human epithelial cells and highly virulent in the *Galleria* infection model. The ability of certain *C*. *jejuni* isolates to spread from the gastrointestinal tract, coupled with the ability to invade human epithelial cells and their increased virulence has implications both control in poultry production and in their risk of causing human disease. There is also variation both in the dynamics and localisation of gastrointestinal colonisation between strains.

Much of our knowledge of the biology of *C*. *jejuni* comes from two or three well-defined isolates, with much of our understanding of its infection biology in the chicken derived from studies in conventional or inbred SPF birds. Whilst these data have informed a basic understanding of this pathogen there have been relatively few studies that utilise isolates found in chicken or other foodstuffs, or that use commercial lines of broilers. Isolates from ST21 and ST257 are frequently associated with both the chicken and gastroenteritis and it is of note that the poultry strains 131226 (ST21) and 12262 (ST257) were more frequently isolated from the liver of both broiler breeds than M1(ST137/CC45), an isolate commonly used in experimental infection studies and which is likely to be laboratory adapted due to repeat passage. Although there is strain-to-strain variation, there was extra-intestinal spread in both the fast-growing standard broiler breed A and the slower growing breed B used in improved welfare production systems. In total 37% of breed A and 29% of breed B birds were found to have *C*. *jejuni* in their livers. The high levels of liver contamination do clearly show that extra-intestinal spread to edible tissues is frequent and a considerable risk, particularly given that certain isolates such as 13126 can give rise to high levels of extra-intestinal spread.

Intriguingly the ST21 isolate 13126 is also significantly more invasive into the Caco-2 human intestinal epithelial cell than the other isolates this phenotype may explain why it is the most successful of the isolates in spreading beyond the gut in the chicken. However cell invasion does not correlate completely with the phenotype of extra-intestinal spread as other isolates, notably M1 and 12262 were also capable of extra-intestinal spread. Indeed DBM1 was the least frequently isolated from liver but was originally isolated from deep muscle tissue and able to infect the muscle of a bird experimentally, something that was not found with any other isolate. This suggests that although an invasive phenotype may be advantageous in extra-intestinal spread of *C*. *jejuni*, there are likely to be multiple mechanisms of spread from the intestine to edible tissues including liver and muscle. Given that extra-intestinal spread is both variable in terms of numbers of birds where *Campylobacter* is detected in the liver and that this is often at a low level that can only be detected by enrichment culture, determining such mechanisms will be challenging.

There also appears to be variation in the dynamics of intestinal colonisation. Whilst 13126 colonises both caeca and ileum to a high level at 11 days post infection, it was not detected in breed A through cloacal swabbing but was found in all birds of breed B. All other isolates were found in every bird of both breeds. This raises a number of important questions. Given the variation between breed and strain combinations in both extra-intestinal spread and colonisation following infection and given that these breeds vary in their immune response to *Campylobacter* infection we might consider that some *C*. *jejuni* are better adapted to different environments resulting from for example, variations in microbiota, immune response, production of mucins, both between breeds and at different gut compartments. Colonisation of the ileum by 13126 is greater in breed A than breed B and further evidence of differences in sites and dynamics of colonisation have been shown in co-infection with M1 and 13126, with the former colonising the caeca rapidly but the latter colonising more sites in the gut including the crop, gizzard and small intestine [16]

As discussed above, isolate 13126 shows a relatively strong phenotype for the invasion of human Caco-2 epithelial cells compared to other *C*. *jejuni* isolates. Furthermore 13126 is highly virulent in the *Galleria* insect model causing rapid melanisation of the larvae and a mortality rate higher than other isolates. Previously it had been shown that ST257 isolates displayed greater virulence than ST21 isolates in this model, but we found the reverse to be true. Indeed one may speculate as to whether 13126 also displays increased virulence during human infection. That poultry and human infection are frequently associated with both ST21 and ST257 may indicate that such sequence types have both an advantage during chicken infection and causing disease. Whilst MLST is a proven tool in molecular epidemiology and indeed find,such associations between disease and individual sequence types, it cannot alone predict variation in pathogenicity within a sequence type. From the data we present here it is apparent that our current understanding of *Campylobacter* biology based upon laboratory isolates such as NCTC11168H or M1 may not hold true for other isolates such as those currently found in the ‘field’. Individual isolates like 13126 from genomic complexes such as ST21, ST257 or ST45 that are commonly associated with both poultry and clinical disease may pose both an increased risk of transmission from poultry and then causing more severe disease in those patients infected with these isolates. Such differences in infection biology in both the main reservoir, the chicken, and in humans may have considerable implications for control.

Control of *C*. *jejuni* in the chicken in the UK has recently focussed on reducing surface contamination of carcasses and reducing caecal colonisation. Whilst this may have some impact in reducing foodborne transmission, the data we present here suggest that ‘field’ isolates, including representatives of ST21 and ST257 genotypes, can frequently cause extra-intestinal infection into edible tissues of the chicken. Indeed this may on occasion include muscle tissue as has been previously shown in the field [27, 28], but shown, we believe, for the first time experimentally here. This presents a different risk and may be considerably more difficult to control, particularly by post-slaughter treatments. Furthermore, as some isolates can colonise the upper gastrointestinal tract, this may mean that practices in processing aimed at reducing the risk of spread from the caeca may not control spread from the crop, gizzard and small intestines as effectively. It, such as those used in this study. Such heterogeneity may also impact on survival through poultry processing, the cold chain and ultimately mean some genotypes are both more likely to reach ‘the fork’ and to cause disease in humans. Added to our increased understanding that behaviour of *C*. *jejuni* in the broiler chicken may differ considerably to that in chicken breeds used in many studies, we perhaps need to consider the biology of ‘field’ isolates and their interactions with chicken breeds used commercially than relying simply on a limited number of laboratory strains that do not fully reflect what goes on in the food chain. What is clear is that not all chickens and not all *Campylobacter* are the same.
